# The perinatal androgen to estrogen ratio and autistic-like traits in the general population: a longitudinal pregnancy cohort study

**DOI:** 10.1186/s11689-015-9114-9

**Published:** 2015-06-07

**Authors:** Esha S. L. Jamnadass, Jeffrey A. Keelan, Lauren P. Hollier, Martha Hickey, Murray T. Maybery, Andrew J. O. Whitehouse

**Affiliations:** School of Psychology, University of Western Australia, 35 Stirling Hwy, Crawley, WA 6009 Australia; Telethon Kids Institute, Centre for Child Health Research, University of Western Australia, 100 Roberts Road, Subiaco, WA 6008 Australia; School of Women’s and Infant’s Health, University of Western Australia, Perth, Australia; Faculty of Health Sciences, Curtin University, Kent Street, Bentley, Western Australia 6102 Australia; Department of Obstetrics and Gynaecology, University of Melbourne and the Royal Women’s Hospital, Victoria, Australia

**Keywords:** Androgens, Estrogens, Autistic-like traits, Sex steroids, Cord blood, Perinatal, Autism-Spectrum Quotient

## Abstract

**Background:**

Prenatal androgen exposure has been hypothesized to be linked to autism spectrum disorder (ASD). While previous studies have found a link between testosterone levels in amniotic fluid and autistic-like traits, a similar relationship has not been found for testosterone in umbilical cord blood. However, it may be the net biological activity of multiple androgens and estrogens that influences postnatal effects of prenatal sex steroids. Accordingly, composite levels of androgens (A) and estrogens (E) were investigated, along with their ratio, in relation to autistic-like traits in young adulthood.

**Methods:**

Sex steroid data in umbilical cord blood were available from 860 individuals at delivery. Samples were analyzed for androgens (testosterone, androstenedione, and dehydroepiandrosterone) and estrogens (estrone, estradiol, estriol, and estetrol). Levels of bioavailable testosterone, estradiol, and estrone were measured and used to calculate A and E composites and the A to E ratio. Participants were approached in early adulthood to complete the autism-spectrum quotient (AQ) as a self-report measure of autistic-like traits, with 183 males (*M* = 20.10 years, SD = 0.65 years) and 189 females (*M* =19.92 years, SD = 0.68 years) providing data.

**Results:**

Males exhibited significantly higher androgen composites and A to E composite ratios than females. Males also scored significantly higher on the details/patterns subscale of the AQ. Subsequent categorical and continuous analyses, which accounted for covariates, revealed no substantial relationships between the A/E composites or the A to E ratio and the AQ total or subscale scores.

**Conclusions:**

The current study found no link between the A/E composites or the A to E ratio in cord blood and autistic-like traits in the population as measured by the AQ. These outcomes do not exclude the possibility that these sex steroid variables may predict other neurodevelopmental traits in early development.

**Electronic supplementary material:**

The online version of this article (doi:10.1186/s11689-015-9114-9) contains supplementary material, which is available to authorized users.

## Background

Autism spectrum disorder (ASD) varies significantly in presentation among those affected [1]. It is therefore unsurprising that the etiology of ASD is thought to be similarly heterogeneous and multifaceted in nature. At present, much remains unknown about the factors that may underlie the disorder [2]. Research involving the identification of early markers is integral to improving our understanding of the mechanisms underlying the disorder and is likely to significantly impact intervention and outcomes for affected individuals [3].

Males are approximately four times more likely than females to be diagnosed with ASD [4, 5]. The extreme male brain theory [6] posits that this may be because characteristics of ASD are on the extreme end of a spectrum of male-typical characteristics. A growing literature has investigated the factors that may trigger brain masculinization, in particular, by examining possible prenatal biological influences affecting brain development and later behavior. The organizational hypothesis proposes that prenatal sex steroid exposure affects fetal brain structure and function and consequently influences postnatal behavior [2]. It is proposed that greater exposure to testosterone (one of the most biologically active androgens) during a sensitive developmental period (weeks 8–24 of gestation) may have masculinizing effects on the fetal brain [7] and hence may be a precursor to autistic-like traits. Circulating testosterone measured in blood samples has been found to be significantly higher in male fetuses than in female fetuses in utero [8].

Prenatal exposure to androgens has been demonstrated to affect a wide range of developmental factors [9]. There is a plethora of research in animal studies that has revealed the effects of androgens as a masculinizing agent in both primate and non-primate species [10–13]. Testosterone has primarily been the focus of research due to its biological potency (its ability to exert receptor-mediated changes in cellular or physiological function); however, there is also preliminary evidence that other androgens such as androstenedione (A4) may have masculinizing effects [14]. Dehydroepiandrosterone (DHEA) has also been suggested to be involved with weak masculinization [15]. In humans, evidence on the masculinizing role of androgens has come in part from research on individuals with congenital adrenal hyperplasia (CAH). CAH occurs as a result of a genetic abnormality that triggers an overproduction of androgens in utero. As a result, female fetuses are exposed to elevated androgen levels that fall within or above the male-typical range. These, and other studies, support the idea that elevated levels of androgens can result in brain and hence behavioral masculinization [16], though it has been argued that this research may not take into account social and sample characteristics that might influence these findings [17]. Often, the effects of testosterone on autistic-like traits have been investigated using indirect methods, such as hand digit ratios. The 2D:4D ratio refers to the ratio of the relative lengths of the second and fourth digits. This ratio has been used for over a century as an indirect measure of prenatal exposure to testosterone and estradiol, the primary biologically active sex steroid in females [18]. There is some evidence of this ratio being related to prenatal sex steroid levels in amniotic fluid [19]. It is posited to be related to adult endocrine system regulation traits such as aggression and sexual encounters [20]. Overall, indirect measures such as the 2D:4D ratio do not show consistent associations with autistic-like traits [7, 21]. Furthermore, the validity of this marker as a representation of sex steroid concentrations is still debatable [22]. The digit ratio measure can be considered an insufficiently sensitive proxy for examining prenatal hormonal effects [23].

In contrast, other studies have attempted to examine prenatal testosterone exposure via direct measurement of sex steroids in amniotic fluid, collected by amniocentesis during the second trimester. This procedure typically occurs during a proposed sensitive time period when sex steroids are considered to have organizational effects on brain development [24]. Amniotic fluid testosterone concentrations have been found to be significantly greater in males compared to females [25].

With regard to ASD, a group of studies conducted by Baron-Cohen and colleagues found that high levels of fetal testosterone in amniotic fluid were related to autistic-like traits in children aged 18 to 24 months for boys [26], decreased eye contact in 1-year-old children [27], increased restricted interests in 4-year-old boys [28], reduced empathy in 6- to 8-year-old children for boys [29], and greater autistic-like traits in children aged 6 to 10 [30]. Furthermore, a more recent study in a large pregnancy cohort found a latent steroidogenic factor that included cortisol as well as all hormones in the Δ4 pathway (progesterone, 17α-hydroxy-progesterone, androstenedione, and testosterone) was significantly elevated in the amniotic fluid of males who later received diagnoses of ASD. However, testosterone alone was not found to be related to a later diagnosis of ASD [31]. However, amniocentesis is often carried out in older women or those with other risk factors and so the results may not be representative of the normal population [32].

The literature suggests that there may be multiple sensitive periods during which sex steroids affect brain functioning and structure [33]. This position is further supported by research in animal models, which has found that the effects of sex steroids are not restricted to the first two trimesters [33–35]. Recently, studies from the Western Australian Pregnancy Cohort (the ‘Raine’ cohort) have measured sex steroid levels in umbilical cord blood at birth [36, 37]. Measuring sex steroids in cord blood is useful because it can provide an indication of how sex steroids may play a role in brain development in late gestation. Furthermore, this method may enable more representative assessment of antenatal steroid exposure on a population level [23]. The cord blood analysis also permits direct assessment of circulating fetal steroid concentrations as opposed to amniotic fluid which primarily reflects fetal urinary testosterone excretion [32]. The third trimester is a period where dramatic brain growth occurs and is an important period for neuronal maturation, gyrification, and rapid axonal growth [38]. Umbilical cord sex steroids are known to relate to a range of informative childhood development markers, such as language development [39, 40], internalizing and externalizing behaviors [41], and spatial abilities [42], and are therefore worth examining in relation to autistic-like traits.

Two studies conducted by our group using the Raine cohort cord blood samples showed that high levels of cord blood testosterone were associated with language delay in boys but not girls [40, 43]. In contrast, another study found no relationship between testosterone concentrations and autistic-like traits measured at age 19 or 20 years via the autism-spectrum quotient AQ [44].

Previous studies have focused in general on androgens and testosterone in particular. However, there is evidence to suggest that estrogens, in particular estradiol, may promote masculinization, as has been demonstrated in rodent models [2]. It has been proposed that estradiol promotes cell survival, death, and proliferation in separate brain regions. It may facilitate new dendritic spine synapses in some brain regions but suppress them in others. The effects of estradiol may be mediated in part via epigenetic changes to the DNA and chromatin in processes that are region-specific but are still poorly understood [34].

Though empirical evidence for the effects of estrogens on human neural development is lacking, androgen and estrogen exposure has been postulated to be responsible for a variety of sexually dimorphic neurodevelopmental and behavioral characteristics including reproductive function [35] and immune function [45]. Sex steroids interact with and may enhance or limit the effects of each other [46, 47]. The nature of potential interactions between androgens and estrogens remains to be investigated. Previous studies have investigated the relative amounts of androgens and estrogens in relation to the 2D:4D ratio. One study involved the ratio between testosterone and estradiol [19] while the other looked at the ratio between androgen and estrogen composites [48]. The relative amounts of androgens to estrogens therefore may be worth examining.

The present research therefore sought to extend previous studies by expanding the number of sex steroids assessed through deriving androgen and estrogen composites using the novel method developed by Hollier et al. [23]. Sex steroid composites and their ratio were then used as possible predictors of autistic-like traits in both the general population and a subset of individuals with ASD. Sex steroids were measured in umbilical cord blood collected at birth, and A and E composites and their ratio were calculated. The androgen composite weights the concentrations of testosterone, A4, and DHEA according to their relative biological potencies and binding affinities to sex-hormone binding globulin (SHBG). The estrogen composite weights the concentrations of estrone (E1), estradiol (E2), estriol (E3), and estetrol (E4) in a similar manner. Based on the free hormone hypothesis, bioavailable sex steroid concentrations were calculated for testosterone, estradiol, and estrone. While this method has been criticized on the basis of methodological inconsistency and unproved assumptions [49], it remains the traditional method for examining bioactive sex steroids and is widely accepted [50]. For the present study, ASD diagnostic measures had been collected in childhood and the AQ had been administered in early adulthood.

It is hypothesized that, based on previous masculinization studies, a high A composite (and possibly A to E ratio) would be associated with increased autistic-like traits in the whole sample and that those individuals with a clinical diagnosis of ASD would have particularly high A and possibly high A to E ratios. Furthermore, it was expected that these associations would occur for outcome variables that demonstrate a sex difference.

## Methods

### Participants

The Raine study is a longitudinal investigation of women and their offspring recruited from the public antenatal clinic at King Edward Memorial Hospital or surrounding private clinics, between May 1989 and November 1991. The sample consisted of 2900 women who had a specific gestational age (between 16 and 20 weeks), English language skills, an expectation to deliver at King Edward Memorial Hospital, and an intention to remain in Western Australia to enable future follow-up of their child [51]. By the end of the recruitment period, 2868 live births (96 %) were available for follow-up. Informed consent was obtained from all mothers and offspring who participated. The Human Ethics Committee at King Edward Memorial Hospital and/or Princess Margaret Hospital for Children in Perth approved participant recruitment and all follow-ups of the study families.

### Measures

#### Sex steroid measurement

Mixed arterial and venous umbilical cord blood was obtained at the birth of 860 deliveries from the intensive ultrasound arm of the cohort which consisted of 1415 singleton pregnancies in total. We confirmed absence of detectable maternal contamination by Mendelian concordance analysis of DNA from matched maternal and cord blood in ten randomly selected sample sets from the cohort using the Affymetrix genome-wide human single nucleotide polymorphisms array 6.0. Immediately after delivery, mixed umbilical arterial-venous (UA to UV ratio) cord blood was collected and allowed to clot and the resulting serum was frozen at −80 °C and stored without thawing until the present study was performed. Eight hundred and three cord blood samples (92.3 %) representing 407 male and 396 female infants had sufficient serum (after removal, aliquoting and archiving of 1 ml for future studies) for steroid analysis. In January 2010, these serum samples were thawed and analyzed for sex steroid content.

Liquid chromatography-tandem mass spectrometry (LCMS/MS) was used to measure total testosterone (TT), A4, and DHEA as described by Keelan et al. [37]. E1, E2, E3, and E4 were measured by LCMS after solvent extraction, as described in detail by Hickey, Hart, and Keelan [36].

SHBG was measured by ELISA using a commercial kit (IBL International, Hamburg, Germany) according to the manufacturer’s instructions. All samples were measured in duplicate by a single operator using assay kits from the same batch. The inter-assay imprecision was <4.5 % (*n* = 25). Intra-assay variation was 5.2 % (*n* = 861). Samples with an initial replicate coefficient of variation (CV) of >10 % were reanalyzed [37].

#### Calculation of bioavailable testosterone, estradiol, and estrone

BioT (nmol/L), representing the fraction of total testosterone either free (unsequestered by SHBG) or bound to serum albumin, was calculated using the following formula [37]

$$ BioT = \left[ free\  testosterone\right] + \left[ albumin\mathit{\hbox{-}} bound\  testosterone\right] $$

Free testosterone was calculated using the empirical method and formula described by Sartorius and colleagues [52]. Albumin levels were adjusted using published reference values to take into account the decrease in serum albumin concentrations with gestational age [53]. Bioavailable concentrations of E1 (BioE_1_) and of E2(BioE_2_) were calculated using the method described by Mazer [54] and adjusted accordingly as described in detail by Hollier et al. [23].

#### Composite measures

The calculated composites take into account the biological potency, binding affinity, and unbound proportion of the sex steroids. Each steroid is weighted according to its biological potency and the following formulas are used:

$$ \mathrm{Androgen}\ \mathrm{composite}=\mathrm{BioT}+0.1\left[\mathrm{A}4\right]+0.01\left[\mathrm{DHEA}\right] $$

$$ \mathrm{Estogen}\ \mathrm{composite}={\mathrm{BioE}}_1+\left[Bio{E}_1\right]+0.1\left[{E}_3\right]+0.02\left[{E}_4\right] $$

The formulas used to calculate the composites are explained in detail in Hollier et al. [23]. The A to E ratio was calculated by dividing the androgen composite by the estrogen composite.

#### ASD diagnosis

Diagnoses were recorded at the 5-, 8-, 10-, 14-, and 17-year follow-ups by asking the parent if a diagnosis of ASD had ever been made by health professionals. Diagnoses were obtained based on the consensus of a speech-language pathologist, psychologist, and pediatrician as mandated in Western Australia.

#### Autism-spectrum quotient (AQ)

The AQ was completed by participants at age 19 or 20 years with the exception of adults with a known diagnosis of any intellectual disability or ASD (due to ethical concerns). The AQ is a 50-item self-report questionnaire designed to measure autistic-like traits in the general population (Baron-Cohen et al. [55]). The items were scored on a 1–4 scale that has been shown to retain more information (see [56]) than the dichotomous scoring described by Baron-Cohen et al. [55]. In contrast to the subscale structure proposed by Baron-Cohen et al. [55], factor analyses of the AQ have identified varying factor structures [56–58]. In the current study, we divided items into the three subscales identified in a previous study of Western Australian young adults [59]: social skills (13 items), details/patterns (7 items), and communication/mindreading (8 items). For the current data set, internal reliability (α) for the scales was 0.63 for communication/mind-reading, 0.78 for details/patterns, and 0.85 for social skills. Scores for the items within each subscale were then summed to provide a quantitative measure of that particular autistic-like trait, with higher scores representing more autistic-like traits.

#### Sample characteristics

A number of additional variables were recorded at various time points. Sociodemographic variables (maternal age at conception, maternal education, family income) were recorded at 18 weeks’ pregnancy, antenatal variables (maternal smoking and alcohol consumption during pregnancy) at 34 weeks’ pregnancy, and obstetric variables (gestational age, offspring gender, parity, Apgar scores 5 min after delivery) at birth. Proportion of optimal birth weight was also calculated based on the ratio of the observed birth dimension to the optimal birth dimension for that individual neonate [60]. This provided a measure of the appropriateness of fetal growth. Further investigation of these variables was carried out to assess the representativeness of the individuals whose cord blood sex steroids and AQ data were available compared to the broader cohort with sex steroid data.

#### Statistical analysis

As a preliminary check, a principal component analysis with a direct oblimin rotation was conducted on the seven sex steroid variables to determine if the androgens and estrogens formed two separate factors. As expected, the variables loaded on two components with the estrogens loading on the first component (range of loadings 0.76 to 0.88) and the androgens on the second (range 0.63 to 0.72), with cross loadings no higher than 0.46. The literature suggests that prenatal testosterone exposure may exert varying effects between sexes [43]. As a result, all subsequent analyses were carried out after segregating by sex.

Our hypothesis was that the A composite (and perhaps the A to E ratio) contributes to between sex differences in autistic-like characteristics. Therefore, independent sample *t* tests were conducted in order to determine if sex differences existed. This was followed by correlational and hierarchical multiple linear regression analyses using the sex steroid variables to predict AQ scores. For the regression analyses, covariates that showed a correlation with the outcome variable (score on relevant AQ scale) at the level of *p* < .2 were included in the first block using the enter method, while each predictor variable (A, E or A to E ratio) was added in the second block.

The data were then investigated categorically to determine whether sex steroid exposure was associated with high scores on the AQ and its subscales. *High* scores were defined as scores in the upper decile of a particular scale. Sex differences were examined via chi-square analyses, and scales that demonstrated a significant sex difference were examined in relation to sex steroid composite values. For these scales, chi-square analyses were conducted separately for each sex by forming quartiles of androgen, estrogen, and A to E ratios and cross-tabulating these with high scores on the particular AQ scale. Any significant difference on any scale was followed up with multivariate logistic regression, following the same two-step procedure outlined for the multivariate linear regression analyses. For all analyses, an alpha level of 0.05 was adopted.

Additional analyses of androgen levels, estrogen levels, and their ratios in individuals with an ASD diagnosis were conducted. *Z* score conversions of these values enabled them to be compared to the wider sample.

## Results

### Sample characteristics

Sex steroid data were available for 860 children. Of these, 705 (82 %) provided additional diagnostic data at the 5-, 8-, 10-, 14-, or 17-year follow-ups. Of those who had sex steroid and postnatal diagnostic data available, 183 of 364 (50.2 %) males and 189 of 341 (55.4 %) females also completed the AQ. Although age was significantly different for males (*M* = 20.10, SD = 0.65) and females (*M* = 19.92, SD = 0.68) who completed the AQ, *t*(370) = 2.64, *p* = .01, this difference was small, Cohen’s *d* = 0.27. A chi-squared analysis was used to compare the characteristics of those who did and did not complete the AQ within the sample of those who had available sex steroid data. Table 1 presents the characteristics of the two groups. The mothers of those who did not complete the AQ were more likely to be younger, less likely to have completed secondary school, smoke cigarettes and drink alcohol while pregnant as well as earn an income less than the government-defined *poverty line* of $AUD 24,000. However, there was no difference between individuals who did and did not complete the AQ in terms of gestational age at birth, proportion of optimal birth weight, parity or Apgar scores 5 min after birth. There was no significant difference between males who did or did not complete the AQ in androgen, *t*(362) = 0.86, *p* = .39, estrogen, *t*(362) = 1.47, *p* = .14, or ratio, *t*(337.07) = 1.21, *p* = .23, composite values. Females who did versus did not complete the AQ did not differ significantly in androgen, *t*(339) = 1.09, *p* = .28, or estrogen, *t*(339) = 0.81, *p* = .42, composite values. However, females who completed the AQ (*M* = 0.0046, SD = 0.0025) had a significantly lower A to E composites ratio than females who did not complete the AQ (*M* = 0.0064, SD = 0.0099), *t*(339) = 2.43, *p* < .05, Cohen’s *d* = 0.25.

**Table 1:** Characteristics of those who did and did not complete the AQ

Categorical variables	Completed AQ (*n* = 372)		Did not complete AQ (*n* = 333)		*p* value
	Number	*n* (%)	Number	*n* (%)	
Maternal age at birth	364		321		<.01
<20		19 (5.2)		32 (10)	
20–24		65 (17.9)		73 (22.7)	
25–29		90 (24.7)		108 (33.6)	
30–34		119 (32.7)		76 (23.7)	
35+		71 (19.5)		32 (10)	
Maternal education at pregnancy	364		321		<.01
Completed secondary school		155 (42.6)		216 (67.3)	
Did not complete secondary school		209 (56.2)		105 (32.7)	
Family income below poverty line	362		319		<.01
Yes		115 (31.8)		149 (46.7)	
No		247 (66.4)		170 (53.3)	
Maternal smoking in pregnancy	364		321		<.05
None		291 (79.9)		224 (69.8)	
1–10 cigarettes daily		42 (11.5)		56 (17.4)	
11+ cigarettes daily		31 (8.5)		41 (12.8)	
Maternal alcohol intake during pregnancy	364		321		<.05
None		199 (54.7)		213 (66.4)	
Once a week or less		141 (38.7)		88 (27.4)	
Several times a week or more		24 (6.6)		20 (6.2)	
Gestational age	364		321		.87
<32 weeks		3 (0.8)		7 (2.2)	
32 to 37 weeks		64 (17.6)		48 (15.0)	
38 to 40 weeks		230 (63.2)		211 (65.7)	
>40 weeks		67 (18.4)		55 (17.1)	
Proportion of optimal birth weight	370		333		.39
<90		117 (31.6)		96 (28.8)	
90 to 110		206 (55.7)		184 (55.3)	
>110		47 (12.7)		53 (15.9)	
Sex	372		333		.11
Male		183 (49.2)		181 (54.4)	
Female		189 (50.8)		152 (45.6)	
Apgar score	364		320		.94
Generally normal		351 (96.4)		309 (96.6)	
Fairly low		13 (3.6)		11 (3.4)	
Critically low		0		0	
Parity	372		333		.51
0		167 (44.9)		159 (47.7)	
1		121 (32.5)		96 (28.8)	
>1		84 (22.6)		78 (23.4)	

### Sex differences in sex steroid composite values and AQ scores

Table 2 shows descriptive statistics for the sex steroid (androgen composite, estrogen composite, and A to E ratio) and AQ variables for males and females. Independent sample *t* tests were used to examine any sex differences after a logarithmic transformation was applied to any variables for which |skewness| >1. The results of these analyses are presented in Table 2. Analysis of the sex steroid composites and ratio revealed that males had significantly higher androgen levels compared to females (*p* < .01) and a higher A to E ratio (*p* < .05); however, there was no significant difference in estrogen levels between sexes.

**Table 2:** Mean (SD) for sex steroid variables and autism-spectrum quotient (AQ) scores

	Males (*N* = 183)	Females (*N* = 189)	*p* value	Cohen’s *d*
Androgen composite	0.45 (0.27)	0.39 (0.16)	.003	0.27
Estrogen composite	98.66 (46.20)	98.84 (45.19)	.998	0.004
Androgen to estrogen ratio	0.0052 (0.0027)	0.0046 (0.0026)	.02	0.23
AQ				
Total	104.49 (10.56)	102.10 (12.26)	.05	0.21
Social skills	24.69 (5.58)	23.66 (5.86)	.08	0.18
Details/patterns	20.15 (4.64)	18.71 (4.34)	.002	0.32
Communication/mindreading	15.02 (2.88)	15.43 (3.50)	.21	0.13

Analyses of the AQ scores revealed that males scored significantly higher than females on the details/patterns subscale (*p* < .01) and there was a trend towards significantly higher scores for males on the total AQ score (*p* = .05). No sex differences were found on the social skills or the communication/mindreading subscales.

### Sex steroid composite values and AQ scores

Spearman’s rank order correlations were conducted to investigate relationships between the sex steroid variables and AQ variables separately for males and females. All correlations were non-significant as displayed in Table 3. Analyses were also conducted according to the original (dichotomous) AQ scoring and five-subscale structure, but again no significant correlations were demonstrated [see Additional file 1].

**Table 3:** Spearman’s correlations (p value) between sex steroid values and scores on the autism-spectrum quotient (AQ) for males and females

	Androgen composite		Estrogen composite		A to E ratio	
	Males	Females	Males	Females	Males	Females
Alternative scoring						
1. Total	0.07 (.33)	0.11 (.13)	0.11 (.13)	0.08 (.30)	−0.02 (.83)	0.03 (.71)
2. Social skills	0.01 (.90)	−0.02 (.76)	0.06 (.41)	−0.05 (.52)	−0.01 (.94)	0.05 (.49)
3. Patterns/details	0.01 (.87)	−0.01 (.92)	0.01 (.91)	0.07 (.31)	0.03 (.69)	−0.03 (.69)
4. Communication/mindreading	0.06 (.46)	0.04 (.56)	−0.01 (.92)	0.06 (.41)	0.02 (.81)	−0.01 (.94)

### Categorical analyses

Chi-square analyses revealed a sex difference in the proportion of individuals with high scores for one AQ subscale only. Males (13 %) were significantly more likely than females (5.3 %) to have a high score (i.e., fall in the upper decile) on the details/patterns subscale, *χ*^2^ = 6.85, *df* = 1, *p* < .01. No other sex differences were observed. Quartile cutoffs for androgen, estrogen, and A to E ratio composite values for males and females are shown in Table 4. Upper decile cutoffs for the subscales of the AQ were ≥118 (total score), ≥32 (social skills), ≥26 (details/patterns), and ≥18 (communication/mindreading). Chi-square analyses revealed that the proportion of high scores on the details/patterns subscale of the AQ did not significantly vary as a function of androgen (*χ*^2^ = 2.62, *df* = 3, *p* = .45), estrogen (*χ*^2^ = .49, *df* = 3, *p* = .92), or ratio (*χ*^2^ = 3.20, *df* = 3, *p* = .36) quartiles in males. Similarly, in females, the proportion of high scores did not vary as a function of estrogen (*χ*^2^ = 1.93, *df* = 3, *p* = .59) or ratio (*χ*^2^ = 2.14, *df* = 3, *p* = .54) quartiles. However, the AQ details/patterns scores did vary significantly as a function of androgen quartiles (*χ*^2^ = 8.52, *df* = 3, *p* < .05, *ϕ* = 0.21) where females who fell in quartile 2 (the second lowest quartile) in androgen composite values were more likely (36.1 %) than the females in any other quartile to have a high score on the details/patterns AQ subscale.

**Table 4:** Sex steroid quartile cutoffs for males and females

	Androgen composite		Estrogen composite		A to E ratio	
	Males	Females	Males	Females	Males	Females
Lower quartile	<0.33	<0.29	<64.15	<65.53	<0.0033	<0.0029
Quartile 2	0.33–0.41	0.29–0.37	64.16–90.14	65.53–90.94	0.0033–0.0044	0.0029–0.0040
Quartile 3	0.42–0.51	0.37–0.46	90.15–127.42	90.95–123.92	0.0045–0.0066	0.0041–0.0053
Upper quartile	>0.51	>0.46	>127.42	>123.92	>0.0066	>0.0053

As a follow-up to the chi-square analysis for females, a binary logistic regression analysis was carried out to ascertain the effect of androgen quartile on the likelihood of scoring high on the details/patterns AQ subscale. The logistic regression model was statistically significant, *χ*^2^(5) = 13.90, *p* < .05. The model explained 11.5 % (Nagelkerke R^2^) of the variance in details/patterns subscale scores and correctly classified 78.1 % of cases. Females whose androgen levels fell into quartile 2 were 0.34 times more likely to have a high score in the details/patterns subscale after controlling for covariates.

### Clinical ASD

Five children had received a diagnosis of ASD. One male and one female had been diagnosed with autistic disorder and three males had been diagnosed with Asperger’s disorder. Table 5 presents sex-specific *Z* scores for the androgen, estrogen, and A to E values of the individuals with ASD. All but one of the *Z* scores indicated that sex steroid values were within one standard deviation of the sex-specific means. The only exception was a male diagnosed with Asperger’s disorder whose estrogen composite value was slightly more than one standard deviation below the mean (*E* = 43.12 nM/L, *Z* = −1.13).

**Table 5:** Cord blood sex steroid raw values and sex-specific Z scores for each individual diagnosed with ASD

Sex		Androgen composite		Estrogen composite		Ratio	
		Raw value (nM/L)	Sex-specific *Z* score	Raw value (nM/L)	Sex-specific *Z* score	Raw value (nM/L)	Sex-specific Z score
Asperger 1	Male	0.41	−0.11	43.12	−1.13	0.0096	0.51
Asperger 2	Male	0.33	−0.48	80.94	−0.31	0.0041	−0.26
Asperger 3	Male	0.28	−0.68	68.60	−0.58	0.0041	−0.25
Autism 1	Male	0.38	−0.26	98.04	0.06	0.0039	−0.29
Autism 2	Female	0.31	−0.48	104.54	0.16	0.0030	−0.35

### Sample attrition

Previous analyses suggest those who completed the AQ may be biased in terms of socioeconomic factors compared to those who did not. As a result, final post hoc analyses examined whether total AQ scores varied according to these factors. Independent sample *t* tests were conducted and it was found that AQ scores were significantly higher, *t*(360) = 2.15, *p* < .05, for those participants whose mother was living below the poverty line during pregnancy (*M* = 105.19, SD = 11.38) compared to those whose mother was not (*M* = 102.39, SD = 11.63). Similarly, total AQ scores were significantly higher, *t*(360) = 1.93, *p* < .05, for the children of mothers who did not complete secondary education (*M* = 104.32, SD = 10.53) compared to the children of mothers who did complete secondary education (*M* = 101.96, SD = 12.56). Analyses of variance showed that AQ scores did not differ significantly as a function of maternal age at conception, *F*(4, 359) = 0.21, *p* = .92, maternal smoking during pregnancy *F*(2, 361) = 0.95, *p* = .39, or alcohol consumption during pregnancy, *F*(2, 361) = 1.83, *p* = .16.

## Discussion

The current study explored the relationship between perinatal sex steroids and autistic-like traits in young adulthood. To assess perinatal steroid exposure, composites of biologically active androgens and estrogens in umbilical cord blood were calculated, along with their ratio. This study is therefore unique in both its biological methodology (composite sex steroid calculation and umbilical cord sampling) and the nature of the longitudinal data collected (sample size and period over which data were collected).

Males demonstrated significantly higher androgen and higher A to E composite ratios than females, though these effects were small. In contrast, estrogen levels did not differ significantly between sexes. The significant sex differences in levels of androgens and A to E composite ratio are consistent with previous studies suggesting that males have elevated androgen levels perinatally compared to females [37] .

In regard to sex differences on the AQ, males scored significantly higher than females on the details/patterns subscale. This contrasts with two previous studies that involved Dutch parent and student samples and a Scottish student sample. These studies showed significantly higher scores for males in the total AQ score and in the social skills subscale but not in the details/patterns subscale [57, 61]. The contrasting results found here may reflect cultural differences between samples. It may also be that the Raine cohort represents a more general population sample as opposed to the student and parent samples involved in the two previous studies.

In contrast to our hypotheses, correlational and regression analyses of continuous variables revealed no significant relationships between sex steroid variables and AQ scores. In other words, composite indices of concentrations of androgens and estrogens that took into account a wider range of biologically active sex steroids, as well as their ratio, did not relate to the total AQ score or any of the three subscales. Interestingly, when the data were analyzed categorically, inclusion of covariates in the logistic regression resulted in a small effect being found for females, whereby those whose levels of androgens were in the second lowest quartile scored significantly higher than those who scored in the other quartiles on the details/patterns subscale. This result, however, is likely to be spurious given (a) the lack of sex differences found in the outcome measure, (b) the large number of analyses conducted, and (c) that animal models generally demonstrate linear relationships between androgens within the normal range and behavior. Therefore, the overall pattern of results indicate that the perinatal composites of androgens and estrogens as well as their ratio are not significantly related to autistic-like traits at age 19 and 20 in the general population. This further supports the null results found by Whitehouse et al. (2012) where testosterone concentrations were found to not significantly predict AQ scores in the Raine cohort.

It was hypothesized that a high A composite (and possibly A to E ratio) would predict higher scores on the AQ and that those individuals with a clinical diagnosis would have particularly elevated A composites (and perhaps A to E ratios). In the current cohort, five participants had a known diagnosis of ASD. Contrary to expectations, those participants with an ASD diagnosis did not, overall, display atypical sex steroid values with all but one falling within one standard deviation of the mean for the wider sample. Hence, our findings did not support our primary hypothesis.

There are several possible explanations for the lack of relationship between cord blood sex steroid measures and self-reported autistic-like traits on the AQ. First, it may be that prenatal sex steroids exert organizational effects predominantly during the theorized *sensitive period* that occurs in early gestation (weeks 8–24). Research conducted by Baron-Cohen and colleagues as part of the Cambridge Fetal Testosterone Project has so far demonstrated several relationships between early social markers and prenatal testosterone levels obtained via amniocentesis [27–30]. However, organizational effects may occur during several periods, and furthermore, these periods may change depending on the area of development in which the effects are exerted.

Perhaps the sex steroid levels and interactions that affect aspects of brain development leading to certain autistic-like traits (e.g., those such as early social behaviors) occur early in gestation whereas sex steroids may influence other neurodevelopmental characteristics (e.g., language development) later in gestation. For example, previous studies have found relationships between perinatal testosterone in cord blood and delay in early language development in boys [40, 43]. There is neural evidence to support the development of brain regions in the third trimester of pregnancy that are central to language. During this period, the appearance and growth of the gyri associated with the posterior and frontal areas, including perisylvian structures, occurs [62, 63]. These structures, including the planum temporal, pars triangularis, and the inferior frontal gyrus, are suggested to relate to language function [64, 65]. Furthermore, postmortem [66–68] and neuroimaging [69, 70] studies have found atypical perisylvian structures among children with developmental language difficulties. Therefore, the neurodevelopmental influence of steroids in the perinatal period may be informative for language development.

Future investigation could further delineate the periods of gestation in which sex steroids may play a part in different aspects of brain development and determine how we might go about measuring the factors that contribute to this development in a non-intrusive and safe manner in order to be able to relate these factors to behavior later in life.

Another potential explanation for these results is that the extent to which sex steroids are biologically active is not solely determined by potency and amount. The distribution and functionality of androgen receptors present in the fetus may also be important as receptors are responsible for the binding and translocation of androgens to the nucleus of cells. Furthermore, the functionality of these receptors is determined by the androgen receptor gene which affects the sensitivity of the fetus to androgens [71]. Studies have found that defects in this gene can result in complete or partial androgen insensitivity whereby complete insensitivity results in genotypic males presenting as phenotypic females [72, 73]. The cysteine, adenine, and guanine (CAG) repeat sequence in the androgen receptor (AR) gene, located at Xq11-12, has been known to affect serum testosterone levels. More repeats have been shown to be related to diminished androgen sensitivity and result in a negative feedback loop that increases androgen production [74]. Studies have demonstrated that AR-CAGn and serum testosterone levels are positively correlated in males [75] and inversely associated in females [76]. Similarly, estrogen uptake relies on the dispersion of estrogen-specific receptors. In addition, other sex steroids such as progesterone and xenoestrogens may have possible prenatal influences on the developing brain [77, 78]. Examining sex steroid receptors, their genetic counterparts, and other possible influencing sex steroids is another important direction for research in understanding the complexities of prenatal brain development.

Those individuals whose mother was living below the poverty line as well as children of mothers who did not complete secondary education had significantly higher AQ scores. This is consistent with a recent population-based study in Sweden where low socioeconomic status was found to be a risk factor for ASD [79]. It may be that socioeconomic factors are a risk factor for genetic and or environmental vulnerabilities related to the broad autism phenotype. It is important to note that the direction of causation is unknown, and it may be that parents with autistic-like traits are less likely to complete secondary education and more likely to live below the poverty line. These parents may have children who are more likely to have autistic-like traits.

When examining sample attrition characteristics, it was found that the mothers of the individuals who did not complete the AQ were socioeconomically disadvantaged and were more likely to be younger, less educated to a secondary level, have an income below the poverty line, smoke cigarettes, and drink alcohol while pregnant, relative to the mothers of those who did complete the AQ. However, sex steroid levels did not differ significantly between males who did and did not complete the AQ. In females, those who completed the AQ did have significantly lower A to E ratios compared to the women who did not complete the questionnaire, but this effect was only small. These results suggest socioeconomic status in the current study may have influenced drop out such that those with lower income were more likely to drop out before completing the AQ.

## Conclusions

In summary, the current study provides no evidence to suggest that perinatal androgens, estrogens, or the A to E ratio sampled via cord blood predicts autistic-like traits in young adulthood. However, it does confirm expectations that higher androgen composite values and higher A to E ratios in males are present perinatally. It may be that different organizational effects occur early in gestation to those of late gestation, and that these are not evident in the circulating steroid profile at delivery. It is important to note that the nature of sex steroid receptors and genetic influences may play a part in brain development and hence the development of autistic-like traits.

## Additional file

Additional file 1:
**Spearman’s correlations (** ***p*** **value) between sex steroid values and scores on the autism-spectrum quotient (AQ), where the AQ has been scored and subdivided according to Baron-Cohen et al. [** 55 **].** Analyses were conducted according to the original (dichotomous) AQ scoring and five-subscale structure and no significant correlations were demonstrated.
