# Supplementary material for: The perinatal androgen to estrogen ratio and autistic-like traits in the general population: a longitudinal pregnancy cohort study
Source: J Neurodev Disord. 2015 Jun 7;7(1):17. doi: 10.1186/s11689-015-9114-9 (PMC4470005; doi:10.1186/s11689-015-9114-9)
Supplement: Additional file 1: — Spearman’s correlations ( p value) between sex steroid values and scores on the autism-spectrum quotient (AQ), where the AQ has been scored and subdivided according to Baron-Cohen et al. [ 55 ]. Analyses were conducted according to the original (dichotomous) AQ scoring and five-subscale structure and no significant correlations were demonstrated. [file 11689_2015_9114_MOESM1_ESM.doc]

**Additional file 1. Spearman’s correlations (p value) between sex steroid values and scores on the Autism-Spectrum Quotient (AQ), where the AQ has been scored and subdivided according to Baron-Cohen et al. [50].**

|  | **Androgen composite** | | **Estrogen composite** | | **A:E Ratio** | |
| --- | --- | --- | --- | --- | --- | --- |
|  | Males | Females | Males | Females | Males | Females |
| 1. Total | .07 (.33) | .11 (.13) | .12 (.13) | .08 (.30) | .02 (.83) | .03 (.71) |
| 2. Social Skills | .06 (.46) | .04 (.61) | .08 (.30) | .04 (.57) | -.03 (.72) | .01 (.93) |
| 3. Imagination | .08 (.30) | .12 (.10) | .18 (.16) | .03 (.64) | -.12 (.11) | .03 (.64) |
| 4. Communication | .10 (.17) | .06 (.43) | .07 (.34) | -.05 (.49) | .01 (.91) | -.05 (.49) |
| 5.Attention Switching | .10 (.20) | .12 (.11) | .06 (.43) | .04 (.64) | .04 (.58) | .04 (.64) |
| 6. Attention to detail | -.03 (.64) | .02 (.75) | -.04 (.63) | .06 (.45) | .05 (.49) | .003 (.96) |
